# Brain Neurons during Physiological Aging: Morphological Features, Autophagic and Mitochondrial Contribution

**DOI:** 10.3390/ijms231810695

**Published:** 2022-09-14

**Authors:** Vladimir Sukhorukov, Alina Magnaeva, Tatiana Baranich, Anna Gofman, Dmitry Voronkov, Tatiana Gulevskaya, Valeria Glinkina, Sergey Illarioshkin

**Affiliations:** 1 Department for Brain Research, Research Center of Neurology, 125367 Moscow, Russia; 2 Department for Histology, Embryology, and Cytology, Pirogov Russian National Research Medical University, 117997 Moscow, Russia; 3 International Medical Faculty, Pirogov Russian National Research Medical University, 117997 Moscow, Russia

**Keywords:** aging, neurons, macroautophagy, chaperon-mediated autophagy, mitochondria

## Abstract

Accumulating data suggest that the brain undergoes various changes during aging. Among them are loss of both white and gray matter, neurons and synapses degeneration, as well as oxidative, inflammatory, and biochemical changes. The above-mentioned age-related features are closely related to autophagy and mitochondria. Therefore, we aimed to reveal the most peculiar morphological features of brain nervous tissue and to characterize the expression of autophagy and mitochondrial immunohistochemical biomarkers in neurons of different human brain zones during aging. Counting the number of neurons as well as Microtubule-associated proteins 1A/1B light chain 3B (LC3B), Heat shock protein 70 (HSP70), Lysosome-associated membrane protein type 2A (LAMP2A), Alpha subunit of ATP synthase (ATP5A), and Parkinson disease protein 7 (DJ1) immunohistochemical staining were performed on FFPE samples of human prefrontal cortex, corpus striatum, and hippocampus obtained from autopsy. Statistical analysis revealed a loss of neurons in the studied elderly group in comparison to the young group. When the expression of macroautophagy (LC3B), chaperon-mediated autophagy (HSP70, LAMP2A), and mitochondrial respiratory chain complex V (ATP5A) markers for the young and elderly groups were compared, the latter was found to have a significantly higher rate of optical density, whilst there was no significance in DJ1 expression. These findings, while preliminary, suggest that both autophagy and mitochondria are involved in neuronal maintenance during aging and could indicate their potential role in adaptive mechanisms that occur in aging.

## 1. Introduction

Accumulating data suggest that the brain undergoes various changes during aging, which is a major risk factor for many neurodegenerative disorders [1]. Among the above-mentioned changes are weight and volume decrease due to loss of both white and gray matter, losing neurons, and the degeneration of myelin fibers, neurites, and synapses. Many changes that occur during aging and neurodegeneration demonstrate similar morphology and could mainly differ quantitatively [2,3].

Over the many years, it has become evident that at the cellular level, brain aging is closely related to oxidative stress, bioenergetic deficiency, accumulation of damaged ultrastructural components, and aggregated neurotoxic proteins [4,5]. These hallmarks could contribute to age-related neurodegenerative diseases, and therefore maintaining a functional pool of neurons depends on mitochondria and autophagy; this makes the investigation of the latter relevant in the development of new biomarkers for neurodegenerative target therapy.

For non-dividing cell types, which neurons belong to, the maintenance of neuronal cytoplasm and cytotoxic protein elimination could be provided by various types of autophagy [6]. Macroautophagy is the main autophagy pathway, which is a protective mechanism that allows cells to function under stress factors such as energy deprivation, hypoxia, lack of growth factors, reactive oxygen species, and DNA damage. Macroautophagy eliminates dysfunctional proteins, whilst its disruption can lead to the impairment of axonal movement, dendrite and axon remodeling, and as a result, a decrease in the plasticity of the nervous tissue [6].

Chaperone-mediated autophagy (CMA), a selective type of autophagy, also plays an important role in neuron functioning and survival during aging. It is characterized by the following: proteins with the KFERQ motif are recognized and then bind with the cytosolic heat shock protein 70 (HSP70). Then, this complex interacts with the lysosome-associated membrane protein type 2A (LAMP2A) to translocate the cargo into the lysosome for hydrolysis [7]. Chaperone-mediated autophagy dysfunction leads to the decreased degradation of misfolded neurotoxic proteins, e.g., tau proteins, β-amyloid in Alzheimer’s disease, α-synuclein in Parkinson’s disease, the mutant protein polyQ-huntingtin in Huntington’s chorea, and the TDP-43 protein in frontotemporal dementia [8,9,10]. Furthermore, CMA is also indirectly involved in mitochondrial quality control by eliminating the dysfunctional protein DJ-1 (PARK7) with the KFERQ amino acid sequence [11,12,13,14].

It has conclusively been shown that both autophagy and mitochondria play a central role in coordinating metabolic processes and cell homeostasis [15]. In turn, damaged organelles could contribute to aging and age-related neurodegenerative disorders by decreased oxidative phosphorylation and increased reactive oxygen species, as well as via proapoptotic signaling, inflammasome activation, and disordered synaptic transmission [16,17].

Both mitochondria and autophagy appear to be fundamental signaling platforms that regulate miscellaneous processes and provide the adequate cell function as a unified system. Whilst cell compartmentalization exists, their interplay coordinates various processes in cells, and the defects in one or the other system contributes to aging and age-related diseases [18].

Consequently, we aimed to reveal the most peculiar morphological features of senile neurons as well as to characterize the expression of macroautophagy (LC3B), chaperon-mediated autophagy (HSP70, LAMP2A), the mitochondrial respiratory chain complex V (ATP5A), and the mitochondrial quality control (DJ1) immunohistochemical biomarkers in the neurons of different human brain zones during aging.

## 2. Results

### 2.1. Common Histological Methods

In our study, statistically significant differences in the number of large neurons in the studied areas of the brain were established between two age groups (Table 1). Based on the obtained results, there was a progressive decrease in the number of neurons in the substantia nigra, corpus striatum, the pyramidal layer of the hippocampus, and layer V of the cortex of elderly people as compared with those of young people.

**Figure 1:**
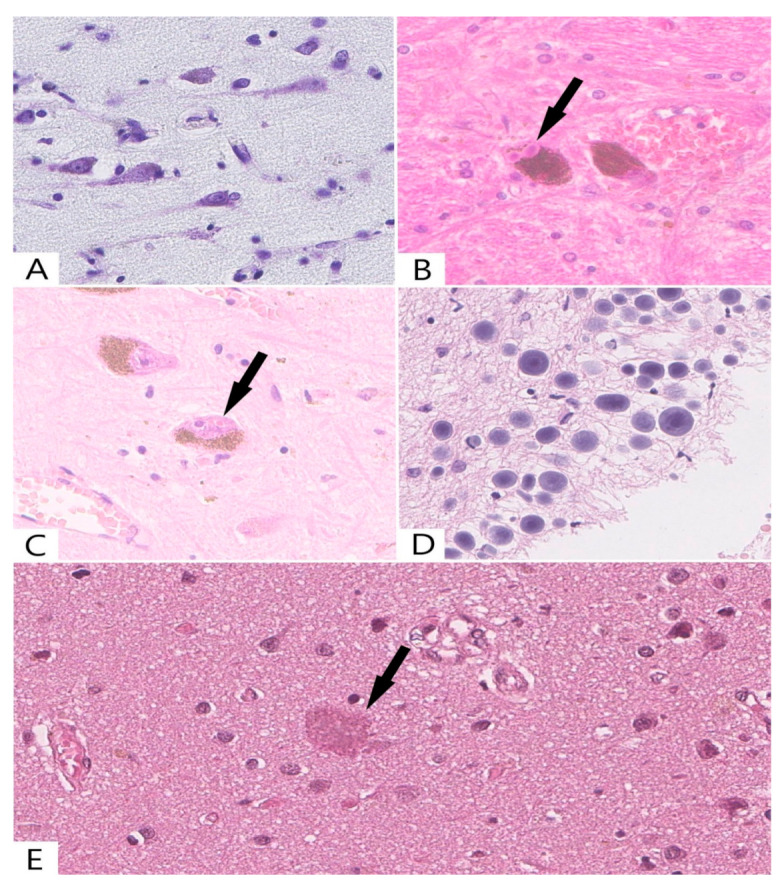
Morphologic changes in neurons during aging. (**A**) Neuronal chromatolysis and “ghost form’’ of neurons; (**B**) Lewy bodies (arrow) with a dense and intensely colored core and a clear amorphous peripheral part in the nigrostriatal neuron; (**C**) Two eosinophilic intranuclear inclusions near the nucleoli (Marinesco bodies (arrow)) in the nigrostriatal neuron; (**D**) Clusters of corpora amylacea (some of them demonstrating a lamellar structure) in the precentral gyrus near subarachnoid space; (**E**) Senile plague (arrow) in the precentral gyrus cortex. (**A**)—Nissl stain, ×400; (**B**–**E**)—Hematoxylin and eosin stain, ×400.

It should be emphasized that senile changes do not only include the death of neurons but also a decrease in their volume, accompanied by a decrease in the amount of Nissl substance in the cytoplasm and a decrease in the size of the nucleus. In this study, neuronal chromatolysis with a transformation into the “ghost form’’ of neurons was found in almost all cases in the group of people older than 75 years (Figure 1A).

Light microscopy revealed small amounts of cytoplasmic and intranuclear inclusions (bodies) in the neurons. Lewy bodies were also found in the cytoplasm of the substantia nigra neurons in two cases from the group of elderly people (Figure 1B). In addition to cytoplasmic inclusions, we also identified intranuclear Marinesco bodies (Figure 1C), which were located in the nigrostriatal neurons of the elderly group (n = 8).

Additionally, in the abovementioned group, clusters of corpora amylacea were detected under the ependyma, the pia mater, and around the intracerebral vessels (Figure 1D), while senile plagues were found in small amounts in the cortex and basal ganglia (Figure 1E).

The results of this part of the work have been described in more detail in a previous publication [19].

### 2.2. Immunohistochemical Analysis of Chaperone-Mediated Autophagy (CMA) Markers

The results of the immunohistochemical analysis showed the increased expression of both the heat shock protein 70 (HSP70) and the lysosome-associated membrane protein type 2A (LAMP2A) in the brain samples from elderly patients as compared with the autopsy material obtained from young people.

**Figure 2:**
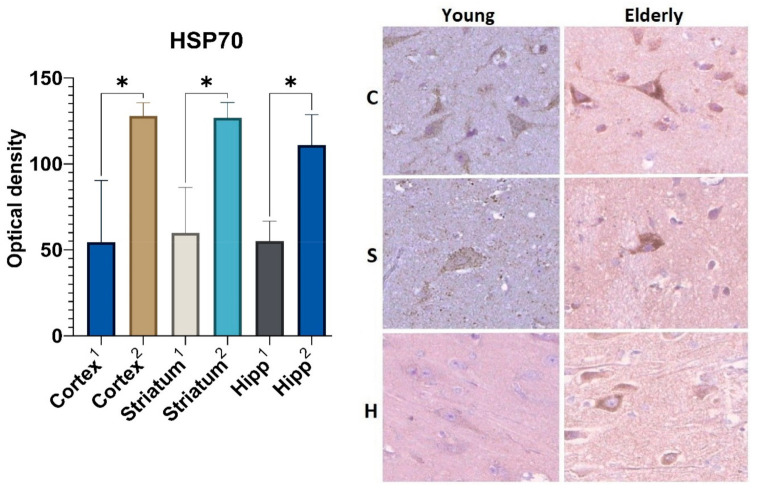
Results of HSP70 immunohistochemical staining: 1—young group, 2—elderly group; C—cortex, S—corpus striatum, H—hippocampus. *—differences are statistically significant (*p* < 0.05). Magnification 400×.

Statistically significant differences in the expression of the HSP70 marker were revealed in all studied areas of the brain from the two groups (Figure 2). The optical density of the HSP70 in large neurons of the precentral gyrus cortex was 2.3-fold higher in those who died over 75 years old (Me = 128) as compared with samples from those who died at a young age (Me = 54.5). A similar tendency was observed in the neurons of the striatum and the pyramidal layer of the hippocampus. Thus, the HSP70 expression in the samples obtained from the elderly group was 2.1 and 2-fold higher, respectively, when compared with samples from people aged 35–45 years.

**Figure 3:**
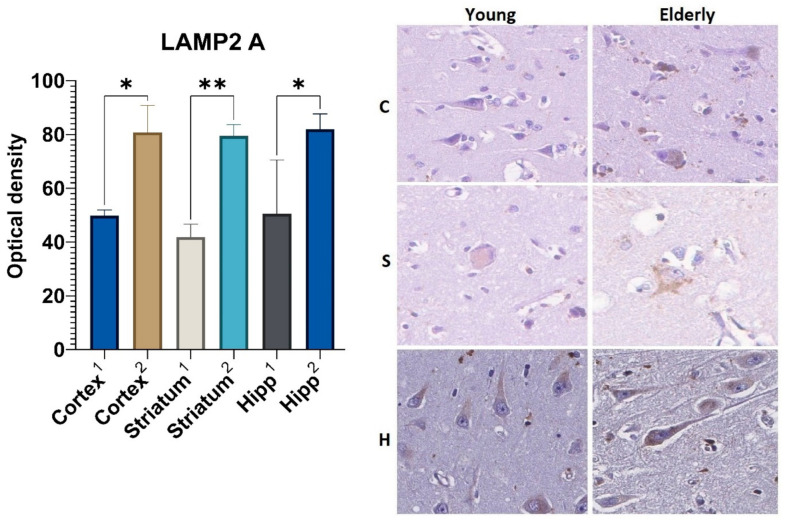
Results of LAMP2A immunohistochemical staining: 1—young group, 2—elderly group; C—cortex, S—corpus striatum, H—hippocampus. *—differences are statistically significant (*p* < 0.05). **—differences are statistically significant (*p* < 0.001). Magnification 400×.

An analysis of the LAMP2A marker also revealed statistically significant differences in all studied areas of the brain according to age (Figure 3). The optical density of this protein in the large neurons of the precentral gyrus cortex was 1.6-fold higher in the group aged 75 years and over as compared with that in the young group (Me = 80.8 and Me = 49.8, respectively). The expression level of the LAMP2A protein in the neurons of the corpus striatum and the neurons of the pyramidal layer of the hippocampus in the samples obtained from the elderly group was 1.8 and 1.5 times higher as compared with those obtained from the control group, respectively.

Moreover, it was found that the optical density of the HSP70 in the samples from the elderly group increased more intensively than the optical density of the LAMP2A. For example, the level of optical density of the HSP70 was higher than that of the control by 59% (in the cortex), 54.6% (in the striatum), and 52.9% (in the hippocampus), while the optical density of the LAMP2A was higher than that of the control by 40.7%, 50%, and 41.8% in similar areas of the brain.

### 2.3. Immunohistochemical Analysis of Macroautophagy Marker LC3B

**Figure 4:**
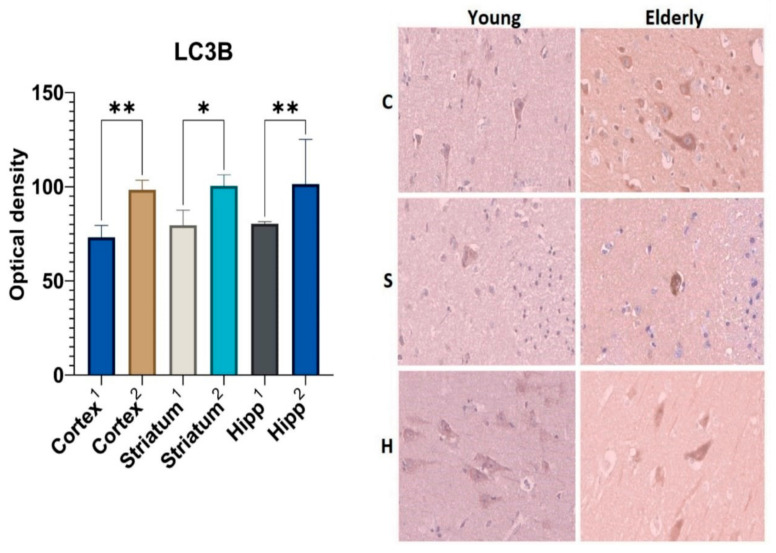
Results of LC3B immunohistochemical staining: 1—young group, 2—elderly group; C—cortex, S—corpus striatum, H—hippocampus. *—differences are statistically significant (*p* < 0.05). **—differences are statistically significant (*p* < 0.001). Magnification 400×.

Analysis of the macroautophagy marker ubiquitin-like protein LC3B revealed statistically significant differences in optical density in all of the studied areas of the brain (Figure 4). The LC3B optical density in the neurons of the precentral gyrus cortex was 1.3-fold higher in the group of elderly people as compared to that of the control group of young people. Similar results were obtained for the neurons of the corpus striatum (Me = 79.6 for the young samples and Me = 100.4 for the elderly) and the hippocampus (Me = 80.6 for the young samples and Me = 101.4 for the elderly).

### 2.4. Immunohistochemical Analysis of Marker of Complex V (ATP5A)

**Figure 5:**
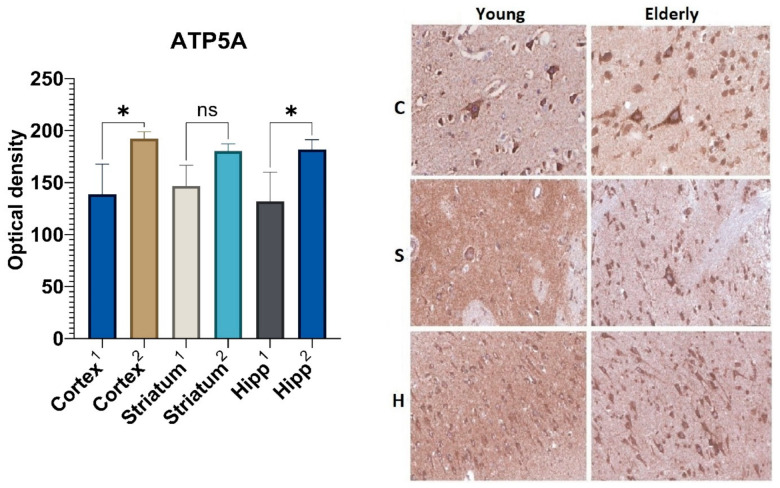
Results of ATP5A immunohistochemical staining: 1—young group, 2—elderly group; C—cortex, S—corpus striatum, H—hippocampus. *—differences are statistically significant (*p* < 0.05). ns—differences are not statistically significant (*p* > 0.05). Magnification 400× for “C” and “S”, Magnification 200× for “H”.

In the fifth layer of the precentral gyrus cortex as well as in the pyramidal layer of the hippocampus, the ATP5A marker’s optical density had statistically significant differences depending on age (Figure 5). In these areas, the optical density of ATP5A in the elderly group was 1.4-fold higher than that in the control group of young people. The expression level of the ATP5A protein in the striatal neurons from samples obtained from the elderly group was 1.2-fold higher as compared to those obtained from the group of young people; however, this result was not statistically significant.

### 2.5. Immunohistochemical Analysis of the Redox-Sensitive Protein DJ1

**Figure 6:**
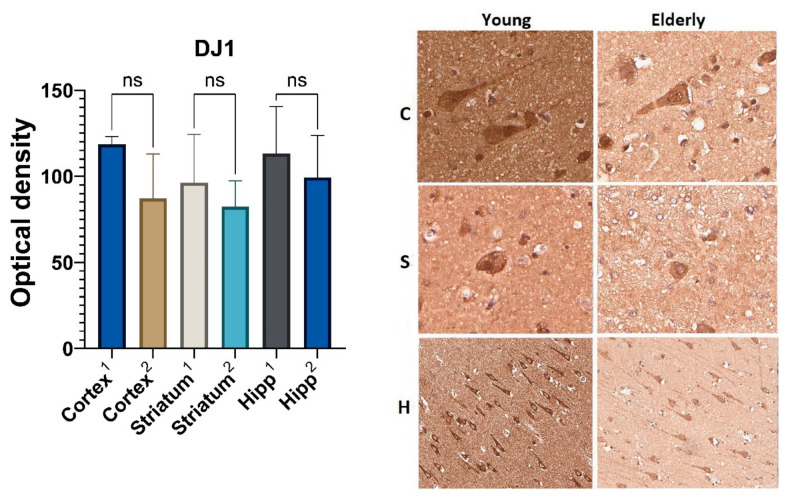
Results of DJ1 immunohistochemical staining: 1—young group, 2—elderly group; C—cortex, S—corpus striatum, H—hippocampus. ns—differences are not statistically significant (*p* > 0.05). Magnification 400× for “C” and “S”, Magnification 200× for “H”.

An analysis of the DJ1 marker’s optical density in the two age groups did not find statistically significant differences. However, in all the studied areas in the group of elderly people, there was a tendency for DJ1to decrease in optical density. Thus, during aging, the DJ1 optical density in the neurons of the precentral gyrus cortex was 26% lower, in the corpus striatum—14%, and in the pyramidal layer of the hippocampus—12% as compared to its optical density in the control group of young people (Figure 6).

In addition, it should be noted that the expression of all the mentioned markers within each age group had no statistically significant differences in the three studied areas of the brain.

## 3. Discussion

For an understanding of the aging process, it is necessary to take into account not only general senile features, but also changes occurring in distinct brain structures. Meanwhile, in publications devoted to quantitative and qualitative senile characteristics, many authors choose the hippocampus as an object of study and rarely single out several structural and functional areas. In the present work, we performed a morphological and morphometric analysis not only of the hippocampus, which is involved in memory consolidation, but also of various structures responsible for complex movements.

Our results confirm that brain areas such as the precentral gyrus, hippocampus, corpus striatum, and substantia nigra are prone to loss of large neurons with age [19,20,21,22,23]. However, it should be noted that some studies demonstrated only a slight decrease in the amount of neurons in different brain areas during aging [20,24,25]. This discrepancy in the obtained results can be explained by several causes.

In this study, the neuronal reduction could, to some extent, be due to cerebrovascular insufficiency as a result of cerebral angiopathy. This is confirmed by the focal change lesions we found in a number of cases due to previously suffered small, deep infarcts, whose development is associated with hypertension. In its turn, the presence of cardiosclerosis as the initial cause of death is evidence in favor of the cardioembolic etiology resulting from previously suffered small superficial infarcts. It should be noted that the impossibility to fully exclude comorbid and age-associated diseases determines the complexity of studying morphological changes caused only by senile manifestations. Differences in the methodological approach to morphometry could also contribute to the discrepancy in the data. Thus, in the above-mentioned studies, stereological analysis was used to assess the results, allowing for a three-dimensional reconstruction of the studied structures.

The obtained data indicate that the morphological examination of the above areas revealed changes similar to neurodegeneration. The most common among them are chromatolysis and hyperchromatosis. At the same time, cytoplasmic Lewy bodies and intranuclear Marinesco bodies were found only in single neurons of the substantia nigra, and senile plaques were detected in small numbers in the neocortex.

Other authors suggest that during physiological aging, there may be an increase in neurotoxic proteins due to both their misfolding and impaired utilization [26]. These processes underlie most neurodegenerative diseases such as Parkinson’s disease, Alzheimer’s disease, and Huntington’s disease [26,27,28]. Previous studies have demonstrated that aggregated protein formation and accumulation are prevented by the chaperone system formed by heat shock proteins such as HSP70. The latter not only provides conformational control of proteins, but also, together with the lysosomal receptor LAMP2A, participates in the elimination of oligomers with the KFERQ amino acid sequence by CMA [29].

However, despite the large amount of data on the functions and mechanisms of this selective type of autophagy, the expression levels of its markers in human brain nerve tissue are scant and contradictory in the current literature. For example, Ye et al. [30] found increased levels of various heat shock proteins in the brain during aging, while Gleixner et al. demonstrated the opposite results [31].

Our study results revealed an increased expression of CMA immunohistochemical markers in the neurons from the elderly samples, which is partially consistent with the other data. Over the past several years, a number of studies have demonstrated an increased level of expression of various heat shock proteins in both brain tissue and myocardium, liver, and skeletal muscle tissue; however, most studies have been performed in models of laboratory vertebrates [29,30,31,32,33,34], while a small number of studies on CMA was performed on biopsy and autopsy material. This fact could be explained by the difficulty of studying changes caused only by senile features and the impossibility to exclude comorbid and age-associated diseases.

The increased levels of CMA markers obtained in the elderly samples may be due to the following. It is known that this type of selective autophagy is one of the regulators of cell homeostasis. Therefore, its expression level can be stress-induced in response to an increased amount of misfolded proteins and reactive oxygen species accumulated over time, for example, as a result of inflammation or hypoxia. The higher optical density of HSP70 compared with that of LAMP2A detected in elderly brain samples was probably caused by the dissociation between the substrate binding and its translocation into the lysosome. Consequently, we can assume that the binding of nonfunctional proteins to HSP70 and co-chaperones occurs faster than their movement into the lysosome lumen via LAMP2A. The insufficiency of the latter probably underlies the dissociation mechanism described above, but this hypothesis requires further investigation. In addition, heat shock proteins have a wider spectrum of functional activity and are involved not only in CMA, but also perform multiple functions in signaling pathways to maintain cellular homeostasis [29].

Little was found in the literature on the question of macroautophagy and its role in the aging of the human brain nervous tissue. As well as the study of chaperone-mediated autophagy, most of the data obtained was from experimental models of laboratory animals.

The marker used in this work to determine the level of macroautophagy, LC3B, was located on the inner and outer layers of the phagophore membrane. During autophagosome maturation, it was removed from the outer surface but remained on its inner surface; therefore, its level may correlate with the number of autophagosomes. However, the interpretation of this protein’s measuring results can be difficult since an increase in the its level may be associated with both the activation of macroautophagy and a decrease in autophagosome–lysosome fusion [26].

Taking into account all of the data mentioned above, it can be assumed that the relatively higher optical density of the chaperone-mediated autophagy markers (HSP70, LAMP2A) compared to the macroautophagy marker (LC3B) probably indicates that the utilization of misfolded proteins that accumulate over time in senile age occurs mainly with the help of a selective type of autophagy.

Both the autophagy system and the mitochondria play an important role in coordinating metabolic processes and maintaining homeostasis, since they are sources of energy and also act as fundamental signaling platforms that regulate many key processes in order to maintain the adequate functioning of the cell as a single system [35]. In turn, mitochondrial dysfunction, which is characterized by decreased activity in the respiratory chain complex enzymes and the impaired function of oxidative phosphorylation, can lead to a decrease in ATP production, and as a result, to a bioenergetic deficit. The last has a significant impact on the functional pool of neurons due to their vulnerability to ATP depletion, and most of their functions directly depend on energy obtained during mitochondrial metabolism [36].

In connection with the above, we studied the marker of the mitochondrial enzyme complex V, ATP synthase. As a result of our study of the elderly brain samples, we could identify the increased optical density of this marker. The obtained results may be associated with an increase in the size of these organelles and the number of their cristae, which arose during the coordination of their fusion and fission processes due to mitochondrial quality control.

In addition, mitochondrial quality control is one of the mechanisms of interaction between mitochondria and chaperone-mediated autophagy, during which proteins containing the KFERQ motif are utilized. One of the proteins containing the KFERQ amino acid sequence is the DJ-1 protein (PARK7), whose main function is to protect mitochondria from damage during oxidative stress [11,12,13,14]. A slight decrease in the optical density of DJ-1 in combination with an increased level of HSP70 in the samples from the elderly group may be due to the increased utilization of its damaged forms by the mechanism of chaperone-mediated autophagy and indirectly confirms the development of oxidative stress in brain neurons during aging. Hence, it could conceivably be hypothesized that the activation of compensatory processes in both mitochondria and autophagy systems is not accompanied by the intensification of mitochondrial quality control. It can therefore be assumed that the abovementioned results may be related to potential mechanisms of mitoptosis and the subsequent death of neurons, leading to a decrease in the number of the latter, which was also observed in our work.

## 4. Materials and Methods

### 4.1. Brain Samples

This study was approved by the Ethics Committee of the Research Center of Neurology (protocol #10-4/20, 27 November 2020). Samples of the elderly human brain from 30 autopsy cases were received. Inclusion criteria were the age of 75 and over, and the main cause of death being pulmonary embolism because of cardiosclerosis. The comparison group consisted of 10 autopsy materials from middle-aged patients (35–45 years old) who died from sudden cardiac death. Among those who died in the elderly group were 10 men and 20 women aged 75–94 years. The average age of the men was 80 ± 6 years, the average age of the women was 85 ± 7 years. Among the middle-aged group, there were 7 men and 3 women. The average age of the men was 40 ± 4 years, the average age of the women was 45 ± 3 years. 

Exclusion criteria were cognitive or motor disorders in anamnesis as well as severe stenosis of precerebral and cerebral arteries. During a macroscopic examination of 13 elderly brain samples, we revealed ischemic cystic lesions and glial scars, and so in these cases, fragments for histological examination were taken from the contralateral hemisphere.

Brain samples were obtained from the cortex of the precentral gyrus, the dorsal part of the corpus striatum (caudate nucleus, putamen, and external segment of pallidum), the substantia nigra, and the hippocampus body no more than 6 h after the death of the organism. Then they were fixed in 10% buffered formalin for 24 h. After paraffin embedding, the samples were cut at 5 µm.

### 4.2. Histological Analysis and Morphometry

For light microscopy, FFPE samples were stained with hematoxylin and eosin, and by using the Nissl method. Digitized specimen slides were obtained using the Aperio AT2 scanning microscope (Leica Biosystems, Muttenz, Switzerland). Neuron chromatolysis and intracellular inclusions were qualitatively assessed. The number of large neurons in the striatum, layer V of the precentral gyrus cortex, the compact part of the substantia nigra, and the pyramidal layer of the hippocampus was determined per unit area (2 mm^2^) with a Leica Qwin 2.7 (Leica Microsystems, Heerbrugg, Switzerland).

### 4.3. Immunohistochemistry Assay

Immunohistochemical staining was performed on a Ventana BenchMark XT stainer (Ventana Medical-Systems, Oro Valley, AZ, USA) using primary antibodies (Table 2). The optical density was estimated in the perikaryon of 150 large neurons, localized in layer V of the cortex of the precentral gyrus, corpus striatum, and pyramidal layer of the hippocampus. Lipofuscin in the cytoplasm was taken into account. To measure the optical density of an 8-bit image, Leica Qwin 2.7 was used.

### 4.4. Statistical Analysis

All statistical analyses were performed using GraphPad Prism 9.3.1 (Dotmatics, San Diego, CA, USA). Statistical significance was analyzed using an analysis of the Student’s *t*-test, and the Mann–Whitney U-test was used to obtain the morphometric results for the neuron number and the Kruskal–Wallis test for the immunohistochemical optical density, as appropriate. Post hoc comparisons were performed using Dunn’s test. Statistical significance was set at *p* < 0.05.

## 5. Conclusions

The results of this study indicate that morphological changes and morphometric characteristics that occur during aging are similar to features of neurodegenerative disorders and mainly differ from them quantitatively. This study has shown that in brain neurons, markers of different types of autophagy, ATP synthase, and mitochondrial quality control undergo change during aging. However, these findings are limited by study sample size. In addition to this, obtained results must be interpreted with caution because the immunohistochemical method indirectly demonstrates the amount of protein in the cell but does not allow for the measurement of its functional activity; therefore, a validation of the obtained results with the help of other biochemical methods is required.

The present study also confirms previous findings and contributes additional evidence that suggests both types of autophagy and mitochondria are involved in proteostasis and in providing an adequate energy level in the cell. Considerably more work using various methods of cell and molecular biology will need to be performed in order to determine the mechanisms of aging.

## Figures and Tables

**Table 1:** Morphometric results on the number of neurons in various brain structures in the studied age groups.

Area of the Brain	Age (Years)	Number of Neurons		*p*-Value
		M ± SD/ Me	SE/ Q_1_–Q_3_	
Substantia nigra pars compacta	35–45	346	328–442	<0.001 *
	≥75	260	282–334	
Corpus striatum	35–45	145	134–193	<0.001 *
	≥75	74	99–125	
Pyramidal layer of the hippocampus	35–45	698 ± 109	34.43	<0.001 *
	≥75	387 ± 116	21.19	
Layer V of the precentral gyrus cortex	35–45	220	188–250	<0.001 *
	≥75	104	123–162	

*—differences are statistically significant (*p* < 0.05).

**Table 2:** Characteristics of primary antibodies.

Antibody	Manufacture	Dilution	Biomarkers Function
HSP70	Invitrogen Cat.#MA3-028	1:100	Heat shock protein 70 involved in chaperone-induced autophagy through protein binding to the KFERQ motif.
LAMP2A	Sigma Aldrich Cat.#HPA029100	1:750	Lysosome-associated membrane protein type 2A, which promotes substrate translocation into the lysosome lumen in chaperone-mediated autophagy.
LC3B	Abcam Cat.#ab192890	1:3000	Microtubule-associated proteins 1A/1B light chain 3B, which is a part of the ubiquitin-like conjugation system and participates in phagophore elongation and autophagosome membrane formation.
ATP5A1	Invitrogen Cat.#43-9800	1:100	Alpha subunit of ATP synthase (V enzyme complex of the inner mitochondrial membrane).
DJ1/PARK7	Invitrogen Cat.#PA5-78362	1:500	Redox-sensitive protein that controls mitochondrial biogenesis and protects mitochondria from damage during oxidative stress.

## Data Availability

Not applicable.
